# Circular RNAs in vascular diseases

**DOI:** 10.3389/fcvm.2023.1247434

**Published:** 2023-09-29

**Authors:** Qiaoyuan Liu, Yaofeng Wang, Tinghong Zhang, Jianwen Fang, Shu Meng

**Affiliations:** Department of Basic Science Research, Guangzhou Laboratory, Guangzhou, China

**Keywords:** circRNAs, vascular diseases, endothelial cells, smooth muscle cells, atherosclerosis, aneurysms

## Abstract

Vascular diseases are the leading cause of morbidity and mortality worldwide and are urgently in need of diagnostic biomarkers and therapeutic strategies. Circular RNAs (circRNAs) represent a unique class of RNAs characterized by a circular loop configuration and have recently been identified to possess a wide variety of biological functions. CircRNAs exhibit exceptional stability, tissue specificity, and are detectable in body fluids, thus holding promise as potential biomarkers. Their encoding function and stable gene expression also position circRNAs as an excellent alternative to gene therapy. Here, we briefly review the biogenesis, degradation, and functions of circRNAs. We summarize circRNAs discovered in major vascular diseases such as atherosclerosis and aneurysms, with a particular focus on molecular mechanisms of circRNAs identified in vascular endothelial cells and smooth muscle cells, in the hope to reveal new directions for mechanism, prognosis and therapeutic targets of vascular diseases.

## Introduction

The vasculature is the most extensive organ system in the body that delivers oxygen and nutrition to all tissues. Vascular diseases are the leading cause of morbidity and mortality worldwide. Therefore, there is an urgent need to identify novel diagnostic biomarkers and therapeutic strategies for vascular diseases.

Circular RNAs (circRNAs) are a type of single-stranded RNA molecules characterized by their covalently closed loop configuration, making them highly resistant to ribonuclease (RNase) digestion. Initially considered as junk RNAs produced by alternative splicing error (1), circRNAs have recently been shown to possess a wide variety of biological functions, including acting as miRNA/RNA binding protein (RBP) sponges, regulating parental genes, and even encoding proteins. This protein-encoding function suggests a potential role of circRNAs in gene therapy. Moreover, circRNAs are also promising biomarker candidates for diseases due to their stable expression, cell type specificity, and relatively high levels in body fluid (2).

Here, we briefly review circRNA biogenesis, degradation, and functions. We summarize circRNAs discovered in major vascular diseases such as atherosclerosis and aneurysm, with a focus on elucidating the molecular mechanisms of circRNAs identified in vascular endothelial cells (VECs) and vascular smooth muscle cells (VSMCs) in the hope to reveal new directions to identify mechanisms, prognosis method and therapeutic targets for vascular diseases.

## Identification of circRNAs

In 1976, plant viroids were first identified as circRNAs (3). Later, circRNAs were observed in the cytoplasm of eukaryotic cell lines through electron microscope (4), which was suspected to be viral RNA genome. In the subsequent years, only a handful of mammalian genes were found to transcribe into circRNAs without clearly defined functions. Over a long period of time, circRNAs are considered as error-spliced junk RNA byproducts without critical biological functions (1).

With the rapid development of next-generation sequencing technologies and bioinformatic tools, thousands of circRNAs have been discovered in diverse species, including virus (5), archaea (6), protists (7), zebrafish (8), mice (9) and human (10). Notably, more than 300,000 circRNAs have been identified in human (11).

Compared with mRNAs and lncRNAs, circRNAs possess several crucial and unique properties. Firstly, they are highly stable compared with linear RNAs. They can resist RNase digestion due to their covalent loop structure (12), thereby escaping from canonical linear RNA degradation. Secondly, the sequences of most circRNAs are highly conserved among species (10, 13). Thirdly, circRNAs expression is tissue specific (14, 15) during development (16). They are highly enriched in the mammalian brain (17) and human platelets (18). Moreover, they are also present in body fluids, including blood, saliva, and urine (19, 20). Fourthly, the expression of circRNAs changes during the transition from physiological to pathological conditions (21, 22).

## Biogenesis of circRNAs

**Figure 1:**
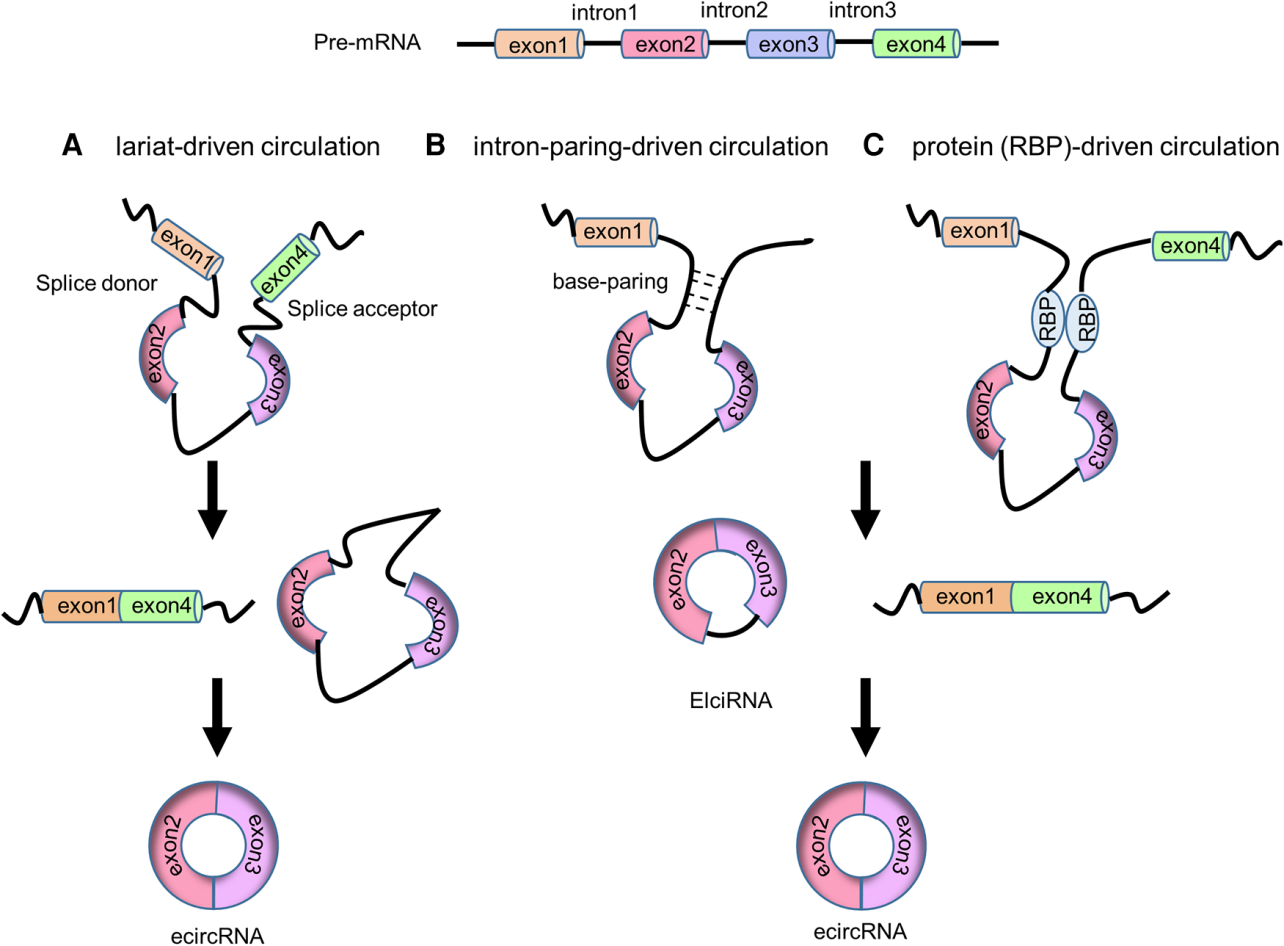
The biogenesis of circRNAs. (**A**) Lariat-driven circulation model. (**B**) Intron-paring-driven circulation model. (**C**) Protein (RBP)-driven circulation model.

**Figure 2:**
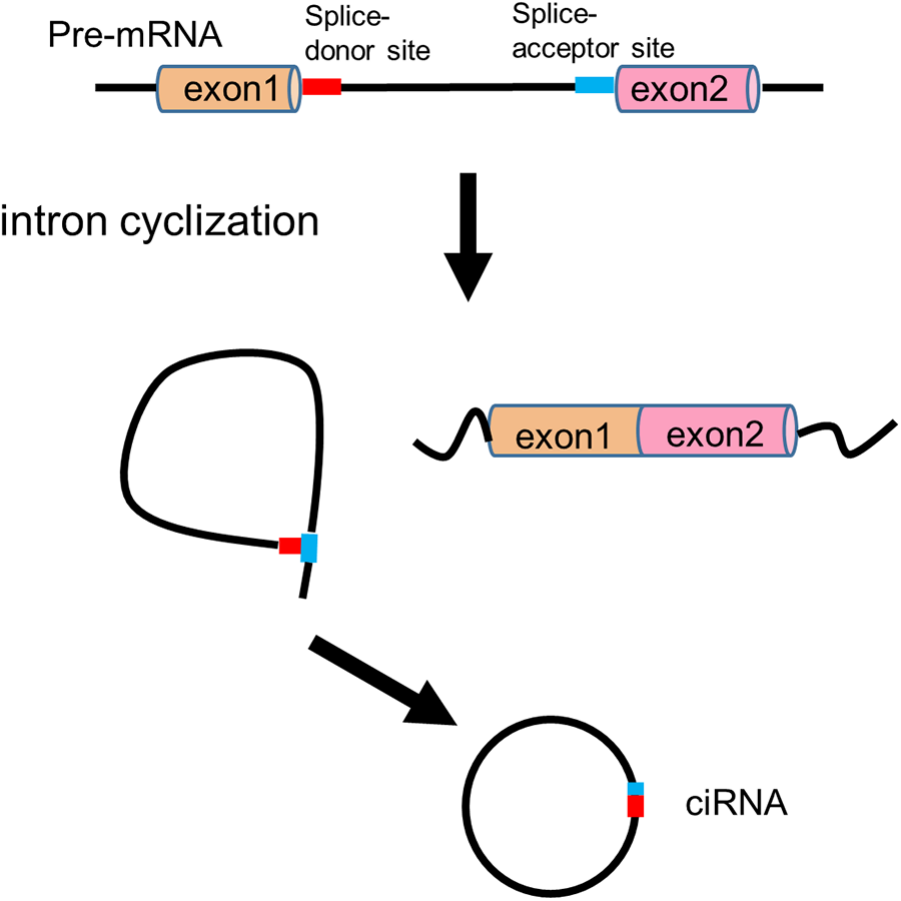
Intron cyclization model of circRNA biogenesis.

In the 1990s, circRNAs were discovered to generate through backsplicing of precursor mRNAs (pre-mRNAs) (9, 23). Both linear mRNAs and circRNAs originate from pre-mRNAs transcribed by RNA polymerase II (Pol II). Linear mRNAs are generated through the spliceosome-involved canonical splicing using splice sites (5'-GC and 3'-AG at introns) (24). In contrast, circRNAs derive from pre-mRNA backsplicing to form a covalent loop structure (10). CircRNAs can generally be divided into three categories based on the sequences they contain: circular intronic RNAs (ciRNAs, circRNAs that solely consist of intron-derived sequence) (25), exon-intron circRNAs (EIciRNAs, circRNAs that contain both exon and intron derived sequences) (26) and exonic circRNAs (ecircRNAs, circRNA that exclusively contain exon derived sequence) (27). Four models of circRNA biogenesis (Figures 1, 2) have been proposed, including lariat-driven circularization (10), intron-paring-driven circularization (10), protein-driven circularization (28), and intron cyclization (25).

In the lariat-driven circularization model, the downstream 3’ splice donor site ligates to the upstream 5’ splice acceptor site to form a 3’ → 5’ phosphodiester bond and generating a lariat consisting of the skipping exons. This lariat then undergoes canonical splicing, releasing the intron and producing an ecircRNA (10) (Figure 1A).

In intron-paring-driven circulation model, the pairing of flanking introns brings the splice sites into close proximity, facilitating backsplicing to create ElciRNAs. Once backsplicing is completed, ElciRNAs can eliminate the introns through canonical splicing, ultimately producing ecircRNAs (10) (Figure 1B). RNA paring can occur at either the repetitive complementary sequence (such as Alu elements) or nonrepetitive complementary sequences (27). These sequences are typically located at the upstream and downstream introns.

In protein-driven circulation model, RBPs can interact with the specific donor and acceptor splice sites, bringing them closer to promote backsplicing, resulting in the formation of EIciRNA and ecircRNA (28) (Figure 1C). For example, RBP Quaking (QKI) has been demonstrated to bind to sites flanking circRNA-forming exons to promote circRNA formation. Insertion of QKI binding sites into linear RNA can induce exon circularization (29).

In intron cyclization model, a consensus motif containing a 7 nt GU-rich element near the 5' splice site and an 11 nt C-rich element close to the branchpoint site can promote the branching enzyme to escape and a debranching failure, ultimately leading to ciRNA formation (25) (Figure 2).

## Functions of circRNAs

### CircRNAs act as miRNA and RBP sponges

CircRNAs, based on the specific sequence motif they contain, can bind to and function as molecular sponges for miRNA and RBP. Through binding and quenching of miRNA/RBP, circRNAs reduce the availability of these molecules to their natural targets, indirectly modulating the expression of the targeted genes.

In 2013, two independent studies reported that circRNA ciRS-7/CDR1as acted as a miR-7 sponge, revealing that circRNAs could function as miRNA sponges (16, 30). CiRS-7/CDR1as exhibits diverse expression patterns in various tissues, with particularly high expression levels in the brain. It originates from the antisense transcript of vertebrate cerebellar degeneration-related 1 (CDR1) and contains 63 miR-7 binding sites. Expression of human ciRS-7/CDR1as in zebrafish impaired midbrain development, resembling the phenotype observed upon miR-7 knockdown, indicating that ciRS-7/CDR1as inhibited miR-7 activity by acting as miR-7 sponge. Additionally, miR-671 could cleave ciRS-7/CDR1as in an Argonaute-dependent manner and release miR-7 (30). Another example is the circRNA known as testis-specific sex-determining region Y (sry), which contains 16 binding sites for miR-138 and serves as a miR-138 sponge (30).

Subsequently, it was revealed that certain circRNAs can act as sponges for multiple miRNAs. For instance, circCHIPK3 can sponge nine different miRNAs through 18 potential binding sites, thereby promoting cell proliferation (22). These miRNA-sponging circRNAs are primarily located in the cytoplasm and can selectively bind to specific miRNAs or groups of miRNAs with their miRNA binding sequences. These binding quenches miRNAs and reduces their binding to target gene transcript, and indirectly enhances the expression of miRNA-targeted genes (16, 30).

CircRNAs can also function as sponges of RBPs and indirectly regulate gene expression. For example, the second exon of RBP muscleblind (MBL/MBNL1) can circularize to form circMbl in flies and humans. This circMbl and its flanking introns could bind strongly and specifically to MBL proteins to decrease Mbl mRNA production (31). Another example is circPABPN1, which shares HuR binding sequences with linear PABPN1 mRNA. CircPABPN1 competes with linear PABPN1 mRNA for HuR binding, an essential RBP recognizing AU-rich elements in the 3' UTR of its target mRNAs. This competition enhances mRNA stability and translation (32).

### CircRNAs regulate parental gene transcription

CircRNAs can regulate their parental gene expression and these circRNAs are primarily located in the nucleus. It was previously reported that intron retention would functionally interfere with the transcriptomes (33), but the exact mechanism remained unclear. So far, both ciRNAs and EIciRNAs, which contain intron sequences, have been reported to locate in the nucleus and regulate their parental gene expression.

CiRNAs, which derive from lariat introns, are abundant in the nucleus and could regulate their parental gene expression through interaction with Pol Ⅱ (25). For instance, the depletion of ci-ankrd52 and ci-sirt7, which are ciRNAs derived from ANKRD52 and SIRT7 introns, leads to a reduction in the transcription efficiency of ANKRD52 and SIRT7 pre-mRNA, respectively (25), suggesting that these ciRNAs exert influence on parental gene transcription by interacting with Pol Ⅱ.

Similarly, EIciRNAs, arising from exon-intron sequences, also possess this regulatory function. EIciRNAs can interact with U1 small nuclear ribonucleoprotein (snRNP), further recruit Pol Ⅱ to its gene promoter region, and enhance parental gene expression (26). For example, knocking down ElciEIF3J and ElciPAIP2 decreased their binding with U1 snRNP and Pol Ⅱ, eventually decreasing EIF3J and PAIP2 transcription (26).

### CircRNAs encode peptides

CircRNAs lack linear mRNA's 5' cap structure and thus cannot use the highly efficient cap-dependent translation mechanism (34) to produce protein. As a result, the majority of circRNAs do not encode peptides. However, they can employ cap-independent mechanisms for translation. Studies have shown that naturally occurring circRNAs can utilize internal ribosome entry site (IRES) elements (35) or m^6^A-dependent translation mechanism (36) to initiate protein synthesis.

In 1986, it was discovered that the hepatitis delta virus possessed a circular RNA genome and could encode proteins in mammalian cells (5). Later, in 1995, the construction of virus IRES into circRNA could initiate the protein coding of ORF through eukaryotic translational mechanism (37). Some naturally occurring circRNAs even contain endogenous IRES sequences that can be recognized by eIF4G2 to initiate translation (35).

Furthermore, the RNA base modification N-methyladenosine (m^6^A) also promotes circRNA translation. The m^6^A reader YTH domain family protein 3 (YTHDF3) can recognize m^6^A modifications and recruit eIF4G2 to initiate translation, a process further enhanced by the methyltransferases METTL3/14 (36). The m^6^A modification can enhance the translation efficiency of circRNAs (38, 39).

### CircRNA degradation

CircRNAs, unlike mRNA with a linear open end, cannot be directly degraded by exoribonuclease. Thus, circRNAs are stable and have a longer half-life in cells. However, the mechanisms underlying circRNA degradation are not fully elucidated. It was initially shown that circRNA *rpsT* could be degraded by RNase E (40, 41). A subunit, Rrp44, of yeast exosome has been shown to harbor the endonuclease activity and can cleave circRNAs; however, the degradation efficiency of Rrp44 for circRNAs is lower compared to linear RNAs (42).

Furthermore, in response to viral infection and activation, the widely expressed cytoplasmic endoribonuclease RNase L can globally degrade circRNAs, thereby contributing to the activation of the double-stranded RNA (dsRNA)-activated protein kinase (PKR) and autoimmunity (43). CircRNAs that carry m^6^A modification can be endoribonucleolytic cleaved by YTHDF2 (m^6^A reader protein)-HRSP12 (adaptor protein)-RNase P/MRP (endoribonucleases) axis (44).

### CircRNAs in vascular diseases

Vasculature systems harbor the heavy duty to deliver oxygen and nutrients to all the tissues and organs in the body. Any disruptions in this intricate system can result in vascular diseases. Two critical cellular components of blood vessels are vascular ECs and SMCs. ECs form the innermost layer of blood vessels and are responsible for secreting various vasoactive substances, such as nitric oxide and angiogenic factors. ECs thus play a pivotal role in regulating vascular tone, maintaining vascular homeostasis, and promoting angiogenesis (45). Vascular SMCs are the main component of the vascular medial layer and are crucial for maintaining vascular structure. The constriction and dilation of VSMC layers regulates vascular tone, blood flow, and blood pressure (46). It has been established that endothelial dysfunction and the phenotypic switch of SMCs contribute to the development and progression of numerous vascular diseases.

With the rapid advancements in next-generation sequencing technologies and bioinformatic tools, researchers have identified dysregulated circRNAs in various vascular diseases, including conditions like atherosclerosis and aneurysms. These circRNAs are appealing due to their stability, presence in body fluids and plasma, and specific tissue expression patterns. Consequently, there is growing anticipation that circRNAs hold the potential to serve as valuable biomarkers for predicting, diagnosing, assessing treatment effectiveness, and determining prognosis in vascular diseases (2). While circRNAs have multiple functions, most of those identified in the context of vascular diseases have primarily been demonstrated to function as miRNA sponges. Functions such as parental gene regulation and peptide encoding have not been reported.

### CircRNAs in atherosclerosis

Atherosclerosis (AS) is a chronic inflammatory disorder characterized by lipid deposition and fibrous cap formation in the arterial wall (47). The initiation of AS is closely associated with low-density lipoprotein (LDL), a particle containing the apolipoprotein B component (48, 49). Endothelial dysfunction and SMC phenotypic switch play critical roles in atherosclerosis initiation and progression (50).

Oxidized LDL (oxLDL) is widely used in *in vitro* cell culture systems to replicate the pathological processes involved in AS initiation. Numerous studies have confirmed that oxLDL contributes to endothelial dysfunction. OxLDL exposure can lead to reduced viability, suppressed migration, apoptosis, inflammatory responses, and oxidative stress in ECs (50). Furthermore, oxLDL can induce a transition in VSMCs from a quiescent, contractile state to a proliferative and synthetic state, both of which play pivotal roles in the formation of atherosclerotic lesions (51).

In recent years, numerous circRNAs have been reported to be dysregulated during the development of AS, with their roles in endothelial dysfunction and SMC phenotypic switching explored. These findings offer a novel direction for potential therapeutic strategies in the treatment of AS, as summarized in Table 1. While many immune cells, such as macrophages and lymphocytes, also play critical roles in AS, they are not discussed here.

**Table 1:** CircRNAs in atherosclerosis.

CircRNAs	Functions	Dysregulation	References
CircKIAA1429	Sponge to miR-1264 to upregulate DNMT1 and activate JAK/STAT pathway	Upregulated in serum exosomes from UA patients	(52)
CircGNAQ	Sponge to miR-146a-5p to upregulate PLK2	Downregulated in senescent HUVECs, aorta tissue of aged mice and blood of older adults	(53)
CircRSF1	Sponge to miR-135b-5p to upregulate HDAC1	Downregulated in oxLDL-treated HUVECs	(54)
CircSPARC	Sponge to miR-328-3p to upregulate TRIM14	Upregulated in oxLDL-treated HUVECs	(55)
CircSMARCA5	Upregulate SRSF1/β-catenin	Downregulated in oxLDL-treated HUVECs	(56)
CircZNF532	Sponge to miR-142-3p to upregulate SIRT3/SOD2 pathway	Downregulated in oxLDL-treated HUVECs	(57)
CircGNB4	Sponge to miR-186-5p to upregulate ROBO1	Upregulated in oxLDL-treated HUVECs	(58)
CircROBO2	Sponge to miR-149-5p to upregulate PAPP-A	Upregulated in oxLDL-treated HUVECs	(59)
CircCHMP5	Sponge to miR-532-5p to upregulate ROCK2	Upregulated in oxLDL-treated HUVECs	(60)
CircNMD3	Sponge to miR-498 to upregulate BAMBI	Downregulated in AS blood samples and oxLDL-treated HUVECs	(61)
CircUSP9X	Sponge to miR-635 to upregulate NLRP3	Upregulated in oxLDL-treated HAECs	(62)
CircNOL12	Upregulate PI3K/AKT/NOS3 pathway	Upregulated in oxLDL-treated HUVECs	(63)
CircHIF1a	Sponge to miR-199a-5p to upregulate SIRT1	Downregulated in oxLDL-treated HAECs	(64)
CircDLGAP4	Sponge to miR-134-5p to upregulate PTPN4	Downregulated in AS patient	(65)
CircFOXO1	Sponge to miR-616-3p to upregulate RFX7	Downregulated in oxLDL-treated HUVECs	(66)
CircTEX14	Sponge to miR-6509-3p to upregulate THAP	Downregulated in AS serum samples and oxLDL-treated HAVSMCs	(67)
CircTM7SF3	Sponge to miR-638 to upregulate ROCK2	Upregulated in AS serum samples and oxLDL-treated HVSMCs	(68)
CircARHGAP12	Sponge to miR-630to upregulate EZH2	Upregulated in the plaque tissue of AS mice and oxLDL-treated MASMCs	(69)
CircUSP36	Sponge to miR-182-5p to upregulate KLF5	Upregulated in AS patients and in oxLDL-treated HUVSMCs	(70)
CircMAPK1	Sponge to miR-22-3p to upregulate MECP2	Upregulated in the mice AS plaque tissues and oxLDL-treated mice VSMCs	(71)
CircPPAPDC1A	Sponge to miR-633 to upregulate CDC20B	Upregulated in oxLDL-treated HVSMCs and the femoral artery wire injury mice	(72)
CircTNPO1	Sponge to miR-181b to upregulate Notch1	Upregulated in AS serum and oxLDL-treated HVSMCs	(73)
CircCHFR	Sponge to miR-370 to upregulate FOXO1 and CCND1; Sponge to miR-214-3p to upregulate STIM1	Upregulated in oxLDL-treated HVSMCs	(74, 75)
CircPTPRA	Sponge to miR-636 to upregulate SP1	Upregulated in AS serum samples and oxLDL-treated HVSMCs	(76)
CircUBR4	Sponge to miR-370-3p to upregulate HMGB1; sponge to miR-107 to regulate ROCK1	Upregulated in AS serum samples and oxLDL-treated HVSMCs	(77, 78)
CircARHGAP32	Sponge to miR-326-3P to upregulate VAMP3	Upregulated in AS serum samples and oxLDL treated HVSMCs	(79)
CircMTO1	Sponge to miR-182-5p to upregulate RASA1	Downregulated in AS serum samples and oxLDL-treated HVSMCs	(80)
CircCOL1A1	Sponge to miR-30a-5p to upregulate SMAD1	Upregulated in AS tissue	(81)
CircTBC1D1	Sponge to miR-183-5p to upregulate FKBPL and BECN1	Downregulated in PDGF-BB-treated HASMCs	(82)
CircANRIL	Bind to PES1 and upregulate p53	Upregulated in carriers of CAD-protective haplotype at 9p21	(83)
CircGRN	Sponge to miR-107 to activate the JAK/STAT pathway; Sponge to miR-377-3p to upregulate AURKA	Upregulated in blood cells of AS patients and oxLDL-treated HVSMCs	(84, 85)

#### EC-related circRNAs

In a study by Wen et al. (52), a comparison of circRNA profiles was conducted in serum exosomes obtained from patients with stable plaque atherosclerosis (SA) and those with unstable/vulnerable plaque atherosclerosis (UA). Their findings revealed a positive correlation between circKIAA1429 (circRNA-0006896) levels in serum exosomes and triglyceride, LDL cholesterol, and C-reactive protein levels in UA patients. Moreover, it was observed that serum exosomes derived from UA patients could upregulate circKIAA1429 expression in HUVECs, reduce microRNA-1264 levels, elevate DNMT1 levels and STAT3 phosphorylation to reduce SOCS3 expression, and notably enhance HUVEC proliferation and migration when compared to both mock and SA groups.

CircGNAQ interacted with miR-146a-5p to upregulate PLK2 expression, inhibiting EC senescence and AS progression (53). AAV-Tie2-circGNAQ, specifically targeting ECs, could curb vascular EC senescence and reduce aortic AS in mice (53). Additionally, circRSF1 overexpression facilitated ECs proliferation and repressed oxLDL-treated HUVE apoptosis and inflammation through the miR-135b-5p/HDAC1 axis (54).

CircSPARC (circ_0004104) is highly expressed in oxLDL-treated HUVECs. Knocking down circSPARC alleviated oxLDL-treated endothelial injury by upregulating miR-328-3p and downregulating TRIM14 (55). Liang et al. found that overexpression of circSMARCA5 (hsa_circ_0001445) activated SRSF1/β-catenin/EMT axis to recover proliferation of oxLDL-treated HUVECs (56). CircZNF532 (circ_0003423) was found to be protective against oxLDL-treated endothelial dysfunction by interacting with miR-142-3p and activating SIRT3/SOD2 pathway (57).

CircGNB4 (circ_0068087) overexpressed in oxLDL-treated HUVECs. CircGNB4 silencing facilitated proliferation of oxLDL-treated HUVECs and reduced oxLDL-treated HUVECs injury through downregulating ROBO1 expression via releasing miR-186-5p (58). CircROBO2 (circ_0124644) promoted oxLDL-treated HUVECs injury by regulating PAPP-A through sponging miR-149-5p (59). Knockdown of circROBO2 suppressed apoptosis and motivated the abilities of cell proliferation and cycle in oxLDL-treated HUVECs (59). CircCHMP5 inhibited cell cycle, proliferation and angiogenesis and facilitated apoptosis to accelerate oxLDL-treated HUVECs injury through upregulating ROCK2 via binding with miR-532-5p (60). CircNMD3 enhanced oxLDL-treated HUVECs proliferation but restrained apoptosis by upregulating BAMBI expression via regulating miR-498 (61). CircUSP9X (circ_0090231) was increased in oxLDL-treated HAECs. Knockdown of circUSP9X could reduce oxLDL-treated HAECs injury and pyroptosis and enhanced their viability through miR-635/NLRP3 axis (62). Inhibition of circNOL12 (hsa_circ_0004543) facilitated HUVECs proliferation, migration, and invasion, significantly reducing their apoptotic rate following oxLDL treatment (63). CircNOL12 knockdown activated PI3K/AKT/eNOS pathway in oxLDL-treated HUVECs to participate in angiogenesis (63).

CircHIF1ɑ (hsa_circ_0032139) promoted HAECs proliferation, motility, and neovascularization through binding with miR-199a-5p to upregulate SIRT1 expression (64). CircDLGAP4 facilitated oxLDL-treated HUVECs proliferation and autophagy, inhibited apoptosis and inflammation, and aggravated dysfunction via interacting with miR-134-5p to increase the expression of PTPN4 (65). CircFOXO1 (hsa_circ_0030042) promoted oxLDL-treated HUVECs proliferation, suppressed apoptosis, and decreased inflammation by targeting with miR-616-3p to upregulate RFX7 levels (66).

#### SMC-related circRNAs

CircTEX14 (hsa_circ_0107197) overexpression attenuated oxLDL-treated HASMCs proliferation and promoted apoptosis via regulating miR-6509-3p/THAP1 axis (67). CircTM7SF3 (hsa_circ_0007478) facilitated proliferation, migration and invasion in HVSMCs through ROCK2 upregulation by binding with miR-638 under oxLDL treatment (68). CircARHGAP12 targeted miR-630 to upregulate EZH2 expression, thereby contributing to the oxLDL-treated proliferation and migration of MASMCs (69). In HUVSMCs, knockdown of circUSP36 modulated oxLDL-treated injury via interacting with miR-182-5p to reduce the expression of KLF5 (70).

CircMAPK1 (mmu_circ_0000668) promoted the proliferation and migration of VSMCs through upregulating MECP2 expression via sponging to miR-22-3p (71). CircPPAPDC1A (hsa_circ_0008896) accelerated AS by enhancing the proliferation, migration, and invasion of VSMCs via binding with hsa-miR-633 to upregulate the expression of CDC20B (72). CircTNPO1 (hsa_circ_0072951) expression levels were significantly increased in the serum of AS patients compared with control. CircTNPO1 promoted the oxLDL-treated proliferation and migration of VSMCs through the miR-181b/Notch1 axis (73). CircCHFR (hsa_circ_0029589) modulates VSMCs phenotypic change. Knockdown of circCHFR suppressed VSMCs proliferation and migration via miR-370/FOXO1/cyclin D1 pathway in AS (74). Downregulation of circCHFR inhibited the proliferation, migration, and invasion of VSMCs by modulating miR-214-3p/STIM1 axis (75).

CircPTPRA expression was upregulated in serum of AS patients and oxLDL-treated VSMCs. CircPTPRA promoted VSMC proliferation and inhibited cell apoptosis through repressing miR-636 to upregulate SP1 (76). CircUBR4 (circ_0010283) interacted with miR-370-3p to upregulate the expression of HMGB1 and regulated the viability and migration of oxLDL-induced VSMCs (77). In addition, circUBR4 acted as miR-107 sponges as well. Blocking circUBR4 could attenuate oxLDL-induced excessive proliferation, migration, and cell cycle progression in HUVSMCs through miR-107/ROCK1 axis (78). CircARHGAP32 (circ_0002984) regulated oxLDL-induced VSMCs proliferation, migration, and inflammation by modulating miR-326-3P to upregulate VAMP3 in AS (79). CircMTO1 expression was decreased in serum of AS patients. CircMTO1 suppressed oxLDL-treated proliferation and migration of VSMCs through increasing RASA1 expression via miR-182-5p sequestration (80). CircCOL1A1 exacerbated VSMCs phenotype switch through miR-30a-5p/SMAD1/TGF-β axis (81). CircTBC1D1 (hsa_circ_0001402) promoted FKBPL expression by targeting miR-183-5p to suppress VSMC proliferation and migration. Additionally, circTBC1D1 enhanced VSMC autophagy by binding with miR-183-5p to increase BECN1 levels (82).

Besides miRNA sponging, circRNA has also been reported to bind with proteins in SMCs. CircANRIL impaired exonuclease-mediated pro-rRNA processing and ribosome biogenesis in VSMCs and macrophages by binding to 60S-ribosome assembly factor PES (83). CircANRIL induced nuclear stress and p53 activation, further induced apoptosis, and inhibited proliferation of VSMCs (83).

#### EC and SMC-related circRNAs

CircRNAs have been reported to regulate both EC and SMC functions. Overexpression of circGRN (circ_0044073) promoted the proliferation of HUVECs and HUVSMCs by interacting with miR-107 and activating the JAK/STAT (84) signaling pathway (85). CircGRN enhanced oxLDL-treated VSMC dysfunction by serving as miR-377-3p sponge to increase AURKA expression (86).

### CircRNAs in aneurysm

Aneurysm refers to the local or diffuse dilation or bulge of a blood vessel (87). The thin and weakened vessel wall is more susceptible to dissection or rupture. Most aneurysms are asymptomatic, but the rupture of aortic aneurysms (AA) or intracranial aneurysms (IA) can be life-threatening (88, 89). Although the molecular mechanism of aneurysm formation is not completely understood, dysregulation of VSMCs and aorta wall matrix degradation are critical pathological changes in AA development (90). This often involves the transition of VSMCs from a contractile phenotype to a proliferative and inflammatory one. Furthermore, the destruction of the extracellular matrix (ECM) is a significant feature of AA, mediated by an imbalance between metalloproteinases (MMPs) and tissue inhibitors of metalloproteinases (TIMPs).

#### AA-related circRNAs

CircRNAs have been reported to regulate SMC proliferation and apoptosis, mainly through their regulation of miRNA expression or miRNA sponge functions (Table 2). CircBTBD7 (hsa-circ-000595) was increased in AA tissue as well as in hypoxic aortic SMCs. Knockdown of circBTBD7 could increase miR-19a expression and reduce hypoxia-induced apoptosis of VSMCs (91). CircCCDC66 was upregulated in AA. Depletion of circCCDC66 enhanced VSMC proliferation and inhibited apoptosis via acting as a miR-342-3p sponge to promote CCDC66 transcription (92). CircChordc1 promoted the VSMCs contractive phenotype and enhanced their growth by vimentin degradation and GSK3β/β-catenin signaling activation, extenuating vascular wall remodeling, and reversing aneurysm progression (93). CircCDR1as served as an inhibitor of miR-7, leading to increased expression of the miR-7 target CKAP4. This promotes the proliferation and reduces the apoptosis of VSMCs (94). CircCBFB functioned as a sponge for miR-28-5p, releasing GRIA4 and LYPD3 from miR-28-5p suppression to promote VSMC growth (95). CircEIF2S2 (hsa_circ_0092291) reduced angiotensin II-induced damage in HAVSMCs by serving as a miR-626 sponge and upregulated COL4A1 expression (96). CircTMEM189-UBE2V1 (hsa_circ_0002168) interacted with miR-545-3p to upregulate CKAP4 levels, facilitating proliferation and restraining apoptosis in VSMCs (97).

**Table 2:** CircRNAs in aneurysm.

Disease	CircRNAs	Functions	Dysregulation	References
Aortic aneurysm	CircBTBD7	Sponge to miR-19a	Upregulated in AA tissue and hypoxic HASMCs	(91)
	CircCCDC66	Sponge to miR-342-3p to upregulate CCDC66	Upregulated in AA mice and Ang II-treated HVSMCs	(92)
	CircChordc1	Upregulate GSK3β/β-catenin signaling	Downregulated in huamn AA tissues and Ang II-treated and CaCl_2_-induced AA mice	(93)
	CircCDR1as	Sponge to miR-7 to upregulate CKAP4	Downregulated in AA tissues	(94)
	CircCBFB	Sponge to miR-28-5p to upregulate GRIA4 and LYPD3	Downregulated in AA tissues	(95)
	CircEIF2S2	Sponge to miR-626 to upregulate COL4A1	Downregulated in AA tissues and Ang II-treated T/G HAVSMCs	(96)
	CircTMEM189-UBE2V1	Sponge to miR-545-3p to upregulated CKAP4	Downregulated in AA tissues	(97)
	CircRBM33	Sponge to miR-4268 to upregulate EPHB2 and downregulate TIMP2	Upregulated in AA tissues and Ang II-treated VSMCs	(98)
	CircFNDC3B	Sponges to miR-143-3p to upregulate ADAM10	Upregulated in AA tissues and Ang II-treated HVSMCs	(99)
Intracranial aneurysm	CircARFIP2	Sponge to miR-338-3p to upregulate KDR	Downregulated in the arterial wall tissues of IA patients	(100)
	CircLIFR	Sponge to miR-1299 to upregulate KDR	Downregulated in artery wall tissues and ASMCs of IA patients	(101)
	CircDOCK1	Sponge to miR-138 to upregulate KDR; Sponge to miR-502-5p to upregulate GREM1; Sponge to miR-409-3p to upregulate MCL1	Downregulated in artery wall tissues and VSMCs of IA patients and in H_2_O_2_-treated HBVSMCs	(102–104)
	CircATL1	Sponge to miR-455 to upregulate SIRT5	Upregulated in IA tissues	(105)
	CircIRAK3	Upregulate OPN, YAP1, MMP2 and MMP9	Upregulated in RIA tissues and HBVSMCs	(106)
	CircIGF2BP3	Sponge to miR-183-5p to upregulate MOP	Upregulated in the ECs of IA patients	(107)
	CircRanGAP1	Sponge to miR-877-3p to upregulate MOP	Upregulated in the ECs of IA patients	(107)
	CircITGAL	Biomarker	Downregulated in blood of IA patients	(108)
	CircPGAP3	Biomarker	Upregulated in UIA with AWE	(109)
	CircDUS2	Biomarker	Upregulated in IA tissues	(110)

CircRNAs have also been reported to regulate ECM degradation (Table 2), suggesting their critical involvement in AA. Wang et al. identified 65 differentially expressed circRNA in abdominal AA tissues and found that circRBM33 was upregulated in AA samples and angiotensin II stimulated VSMCs. CircRBM33 overexpression increased MMP2 expression and reduced TIMP2 expression, leading to ECM degradation. CircRBM33 acting as miR-4268 sponges to upregulate EPHB2 and inhibit TIMP-1 expression to mediate ECM degradation (98). Circ-FNDC3B (hsa_circ_0006156) enhances cytotoxicity in VSMCs triggered by angiotensin II, partly through its role as a miR-143-3p sponge and by upregulating ADAM10 (99).

#### IA-related circRNAs

Hemodynamic disturbances, gene changes, arterial wall degeneration, aging, and infection have been reported as risk factors for IA (111). CircARFIP2 (hsa_circ_0021001) promoted the proliferation, migration, and invasion of human umbilical artery SMCs (HUASMC) by increasing kinase inserts domain receptor (KDR) expression via interacting with the miR-338-3p (100). CircLIFR enhanced the proliferation, migration, invasion, and inhibited apoptosis of HUASMC via binding with miR-1299 to upregulate KDR expression (101). Depletion of circDOCK1 (circ_0020397) involved in decreasing of VSMC proliferation by reducing KDR expression in IA via binding with miR-138 (102). In addition, circDOCK1 could promote VSMC viability via miR-502-5p/GREM1 axis (103). CircDOCK1 alleviated the hydrogen peroxide-induced apoptosis and proliferation inhibition of human brain microvascular SMCs (HBVSMC) by interacting with miR-409-3p to upregulate MCL1 (104). CircATL1 was found overexpressed in IA patients. CircATL1 silencing inhibited VSMCs migration, proliferation and contractility through regulating miR-455/SIRT5 pathway (105). Chen et al. analyzed the differentially expressed circRNAs between unruptured IA (UIA) and ruptured IA (RIA) tissues and found that circIRAK3 (hsa_circ_0005505) upregulated in RIA tissues. Knocking down circIRAK3 inhibited the proliferation and migration of HBVSMCs while inducing apoptosis. Depletion of circIRAK3 reduced expression of HBVSMC phenotype switch marker, including OPN, YAP1 and reduced MMP2 and MMP expression (106).

CircRNAs implicated in endothelial dysfunction also play a role in IA. Zhang et al. analyzed circRNA microarray of ECs isolated from RIA and UIA and identified that circIGF2BP3 (circ_0079586) and circRanGAP1 expression were upregulated in RIA. CircIGF2BP3 and circRanGAP1 increased the myeloperoxidase (MPO) expression through binding with miR-183-5p and miR-877-3p, respectively (107). Notably, MPO has been associated with aneurysm rupture and may serve as a biomarker for IA (112).

In a study by Huang et al., 216 IA patients and 186 healthy volunteers were selected to assess the expression of circITGAL (hsa_circ_0000690) in their peripheral blood. They discovered that circITGAL expression was lower in individuals with multiple IAs than in healthy volunteers, indicating its role as a potential biomarker for IA diagnosis and is closely related to the volume of hemorrhage (108). In another study, Wu et al. analyzed circRNA expression profiles in peripheral blood using circRNA microarrays to compare healthy volunteers with patients harboring saccular aneurysm wall enhancement (AWE). This research revealed that circPGAP3 (hsa_circ_0007990) expression upregulated in UIA patients compared with healthy people. And circPGAP3 expression was significantly higher in UIA patients with AWE than those without. Thus, circPGAP3 could be a novel biomarker for UIA (109). Chen et al. analyzed differentially expressed circRNAs between normal superficial temporal arteries and IA samples and found that circDUS2 was upregulated in IA tissues, suggesting its potential role as biomarker (110).

## Conclusion and future perspective

Over the past decade, our understanding of the functions of circRNAs has begun to emerge. Increasing evidence supports the notion that circRNAs, far from being regarded as “junk RNA”, play pivotal roles in a wide array of biological processes. In the context of vascular diseases like atherosclerosis and aneurysms, numerous circRNAs have been identified within EC and SMC, and their involvement in the pathological progression of these vascular diseases has been indicated. Furthermore, some circRNAs have exhibited differential expression levels in the serum or exosomes of patients with atherosclerosis or aneurysms compared to the general population, suggesting the potential utility of circRNAs as biomarkers for these vascular diseases and for predicting disease prognosis.

However, despite these significant strides, the biological functions and molecular mechanisms of circRNAs in vascular diseases remain incompletely understood. To date, the majority of circRNAs reported to play a role in EC and SMC function in atherosclerosis and aneurysms primarily function as miRNA sponges or regulators of miRNAs. In-depth studies using *in vivo* animal models are scarce, and the roles of circRNAs in functions such as binding to RBPs, regulating parental genes, or encoding peptides have yet to be identified. Further research in these areas is needed to unravel circRNA functions in vascular diseases and to exploit their potential therapeutic and diagnostic applications.

Studying circRNA functions at the DNA level, both in terms of gain and loss of function, is historically challenging, as these approaches can inadvertently affect the levels of their linear RNA counterparts. However, recent advancements in RNA circularization techniques, such as the improved PIE (permuted intron exon) methods (113), have demonstrated high efficiency and the ability to synthesize bulk circRNAs *in vitro*. This has opened up new avenues for circRNA research, particularly in overexpression studies, as it allows for manipulating circRNA levels without altering the linear RNA transcript from their parental genes.

Furthermore, due to the inherent stability and longer half-life of circRNAs, *in vitro* synthesized circRNAs containing specific gene open reading frames hold great promise for gene therapy in the treatment of vascular diseases. These synthesized circRNAs can serve as valuable tools for exploring novel therapeutic strategies.

By integrating bioinformatic methodologies, extensive circRNA profiling, and cutting-edge circRNA synthesis techniques, a more comprehensive understanding of the roles played by circRNAs in vascular diseases can be achieved. This enhanced comprehension of circRNA mechanisms will pave the way for the development of innovative therapies for vascular diseases.
